# Real-World Osteoporosis Pharmacotherapy in the UAE: Prescribing Trends, Adherence, and Patient Beliefs

**DOI:** 10.3390/healthcare14091201

**Published:** 2026-04-29

**Authors:** Maryam Abdulrahman Almoosa Alnuaimi, Syed Arman Rabbani, Khulood Ebrahim Ali Alnaeimi, Khalid Abdulaziz Abu Obaid, Syed Sikandar Shah, Mohamed El-Tanani, Aftab Alam

**Affiliations:** 1 RAK College of Pharmacy, RAK Medical and Health Sciences University, Ras Al Khaimah 11172, United Arab Emirates; maryam.alnuaimi@ehs.gov.ae (M.A.A.A.); syed.sikandar@rakmhsu.ac.ae (S.S.S.); eltanani@rakmhsu.ac.ae (M.E.-T.); aftab@rakmhsu.ac.ae (A.A.); 2 Saqr Hospital, Ras Al Khaimah P.O. Box 5450, United Arab Emirates; khulood.alnaeimi@ehs.gov.ae (K.E.A.A.); khalid.obaid@ehs.gov.ae (K.A.A.O.)

**Keywords:** medication adherence, treatment persistence, osteoporosis, prescribing trends, BMQ, MMAS-8

## Abstract

**Background:** Osteoporosis is a chronic bone disease characterized by reduced bone mass and structural deterioration, increasing fracture risk and affecting patients’ quality of life (QoL). Pharmacological treatments are essential in managing osteoporosis; however, suboptimal prescribing patterns and poor medication adherence can limit therapeutic outcomes. This study primarily aimed to assess medication adherence among patients with osteoporosis using the MMAS-8, as well as prescribing patterns and patient beliefs. **Methods:** We conducted a single-center cross-sectional observational study at Saqr Hospital, Ras al Khaimah, UAE, between October 2024 and May 2025, enrolling 300 adults with clinically diagnosed osteoporosis and/or a bone mineral density T-score ≤ −2.5. Data were collected through structured interviews and medical-record review. Medication adherence was assessed using the 8-item Morisky Medication Adherence Scale (MMAS-8), and beliefs about medicines were measured using the Beliefs about Medicines Questionnaire (BMQ). Prescribing patterns were characterized by drug class, dose, and frequency, and prescribing appropriateness was evaluated using prescribed daily dose/defined daily dose (PDD/DDD) ratios based on WHO ATC/DDD standards. Predictors of adherence were examined using univariate and multivariable Firth penalized logistic regression. **Results:** The median age was 70 years (IQR 63–76), 89.0% of participants were female, and 32.0% had a prior fracture history. Denosumab was the most frequently prescribed anti-osteoporotic therapy (59.0%), followed by romosozumab (30.7%), whereas bisphosphonates and parathyroid hormone analogues were infrequently used (2.7% and 4.7%, respectively). Prescribed dosing closely aligned with WHO standards for all evaluated agents. Overall, 40.7% of patients were classified as adherent and 59.3% as non-adherent. Adherence was not significantly associated with age, gender, nationality, fracture history, polypharmacy, or most comorbidities. In contrast, medication beliefs demonstrated a strong relationship with adherence. In multivariable Firth regression, stronger medication concerns were independently associated with lower odds of adherence (adjusted OR 0.033, 95% CI 0.003–0.355; *p* = 0.0049), while having more than two comorbidities was also associated with reduced adherence (adjusted OR 0.076, 95% CI 0.008–0.688; *p* = 0.022). **Conclusions:** In this UAE real-world cohort, osteoporosis pharmacotherapy was dominated by injectable biologic agents and was prescribed in close agreement with standard dosing recommendations. However, medication adherence remained suboptimal. Patient beliefs, particularly treatment-related concerns, emerged as a more important determinant of adherence than demographic or most clinical characteristics. These findings highlight the need for belief-sensitive, patient-centered adherence interventions alongside optimized pharmacotherapy to improve osteoporosis outcomes in routine practice.

## 1. Introduction

Osteoporosis is a long-term disease of the skeletal system resulting in a reduction in mineral density within the bone and a subsequent change in the physical character of the skeleton, thereby increasing the potential for the development of fragile bones. The most common clinical manifestation of osteoporosis are instances of fragility bone fractures, often resulting from minimal amounts of physical stress on the body (e.g., falling from standing height or falling to the ground) [1]. Fragile bones are also associated with the development of significant long-term pain and loss of the ability to carry out daily activities, which substantially reduce quality of life (QoL). Furthermore, osteoporosis-related fractures continue to impose a significant burden both medically and economically on the world’s healthcare systems [2].

Osteoporosis is becoming increasingly well understood and defined in clinical practice due to improvements in its underlying mechanism, diagnostic criteria, and available therapies both pharmacologically and non-pharmacologically [3]. Osteoporosis is estimated to affect over 200 million individuals worldwide. It affects approximately one-third of women and one-fifth of men aged 50 or older who have suffered from an osteoporotic fracture at some point in their lives [4]. The increase in the incidence of these conditions is due to the increase in life expectancy and the growing elderly population. The World Health Organization (WHO) estimates that approximately one billion people over 60 years old (or 13.5% of the global population) will be affected by osteoporosis by 2030, indicating that the prevalence of osteoporosis and other age-associated disorders will continue to rise over time [5].

Many people who have suffered a serious injury due to osteoporosis can experience life-changing consequences and further complications, including long-term disability, loss of independence, or death, which further increase healthcare costs. Within one year of an injury resulting from a hip fracture, the risk of death ranges from 20% to 30%, and less than half of patients regain their ability to perform at pre-injury levels. Even though comprehensive evidence-based guidelines are available, many fracture patients do not receive appropriate treatment for osteoporosis post-injury. Available data show limited numbers of patients receiving post-fracture therapy within one year following a hip fracture in many countries, ranging from 11.5% in Germany to 50.3% in the UK [6]. There is also a significant financial burden associated with fracture management. The cost of treating osteoporotic fractures in Europe in 2020 was projected to be approximately €37 billion, and on a global scale, there is a projection of over 3 million fractures with an estimated cost of $25.3 billion [7,8].

Despite the availability of effective strategies and standard clinical practice guidelines, osteoporosis management in real-world practice remains suboptimal due to limited awareness, poor medication adherence, and underutilization of effective therapies. Osteoporosis is an area where appropriate support can make a meaningful difference through improved patient education, appropriate medication selection, and practical strategies to enhance adherence. Pharmacists are well placed to contribute by identifying patient risks, guiding appropriate medication use, monitoring side effects, and supporting patients in overcoming adherence barriers. Therefore, it is important to understand how osteoporosis medications are prescribed and how consistently they are used by patients in real-world settings.

In order to bridge this gap, it is critically important to investigate prescribing patterns and patient adherence to osteoporosis medications to better understand the differences between recommended guideline-based care and actual clinical practice. Despite the availability of effective therapies and clinical guidelines, there is limited real-world evidence on medication adherence, prescribing patterns, and patient beliefs regarding osteoporosis treatment in the United Arab Emirates (UAE). Therefore, this study aimed to assess medication adherence, prescribing patterns, and patient beliefs among patients with osteoporosis in a real-world clinical setting in the UAE. This study was designed as an exploratory observational study to assess medication adherence and its association with prescribing patterns, patient beliefs, and quality-of-life outcomes.

## 2. Materials and Methods

### 2.1. Study Design and Population

This cross-sectional observational study with both descriptive and analytical components, aimed at exploring associations rather than testing a predefined hypothesis, was conducted over an eight-month time period in Saqr hospital, a secondary care institution located in Ras al Khaimah, United Arab Emirates, from October 2024 to May 2025. This single-center design was selected to allow detailed evaluation of prescribing practices, medication adherence, and patient beliefs within a consistent real-world clinical setting, minimizing variability in treatment approaches. The primary objectives of the study were to assess prescribing patterns, medication adherence using the MMAS-8, and patient beliefs using the BMQ. The inclusion criteria included any adult of either sex diagnosed with osteoporosis who was receiving pharmacological treatment for osteoporosis at this facility during the time of study. The estimated sample size necessary to conduct this study was calculated based on the expected prevalence of non-adherence to medication among patients with osteoporosis. The sample size was initially calculated using a single population proportion formula, assuming a prevalence of medication non-adherence of 50% (to ensure maximum sample size), a 95% confidence level, and a margin of error of 5%, resulting in a minimum required sample size of 228 participants. To enhance the precision of estimates and account for potential variability in subgroup analyses, the final sample size was increased to 300 participants. Participants were included in the study if they were 18 years of age or older, met the criteria for osteoporosis based on clinical evaluation and/or the results from a bone mineral density test, received osteoporosis treatment, and agreed to participate. Osteoporosis diagnosis was based on bone mineral density (BMD) with a T-score ≤ −2.5 and/or clinical evaluation by the treating physician in accordance with routine clinical practice. Participants with significant cognitive impairment and/or severe psychiatric disorders were excluded from the study to ensure the reliability of self-reported adherence and belief assessments, as these conditions may independently affect medication-taking behavior. Pregnant or lactating women were also excluded, as pregnancy and lactation may influence bone metabolism and require different management strategies. Additionally, individuals who expressed no interest or declined to provide consent were not included in the study.

### 2.2. Data Collection and Study Variables

A detailed data collection form was used to collect data for this study. Data were collected through structured interviews conducted by trained research personnel using standardized questionnaires (MMAS-8 and BMQ), which helped ensure consistency and minimize potential information bias. Written informed consent was obtained from all participants prior to their inclusion in the study. Patients were interviewed for approximately 10–15 min by trained research personnel using a structured data collection form, including standardized questionnaires such as the MMAS-8 and BMQ. In addition, patient record were also analyzed. Missing responses were handled according to standard scoring guidelines. The key demographic and clinical characteristics were obtained which include age, sex, comorbidity profile, duration of osteoporosis, and any prior history of fracture. We captured osteoporosis treatment details, including the prescribed anti-osteoporotic medications, route of administration, dosing regimen, duration of therapy, and use of calcium and vitamin D supplementations.

### 2.3. Assessment of Prescription Patterns

Prescribing patterns and medication utilization were assessed according to the predefined study variables. The predefined variables included treatment indication, the anti-osteoporotic agents prescribed, dosing details (dose and schedule), route of administration, and length of therapy. Additionally, we also captured whether therapy was given as monotherapy or combination therapy, and documented the total number of osteoporotic medications and other concurrent medications.

We collected Defined Daily Dose (DDD) and the Prescribed Daily Dose (PDD) data for each prescribed anti-osteoporotic medication included in the study. The WHO Anatomical Therapeutic Chemical (ATC)/DDD classification system was used as the reference for DDD values. Prescribed daily doses were compared with Defined Daily Dose as a standard reference. The prescribed daily dose to defined daily dose (PDD/DDD) ratio, an established indicator of prescribing appropriateness, was calculated to assess alignment with WHO-recommended dosing standards. This ratio reflects the extent to which actual prescribing aligns with WHO-recommended dosing standards.

The predefined parameters adopted from the previously published and authentic literature were used to review the prescribing patterns and medication utilization. These parameters comprised the reason for prescribing the anti-osteoporotic medicine prescribed, routes of administration, the dosing details (dose and schedule), and the treatment duration. Whether the patients received medications as either a single drug or as a combination therapy was also recorded. Beside this, the numbers of different osteoporosis medications and other concomitant medications prescribed were also documented. The Defined Daily Dose (DDD) and the Prescribed Daily Dose (PDD) for each osteoporotic medication were collected and analyzed. The DDDs were identified using the World Health Organization’s Anatomical Therapeutic Chemical Classification System. The prescribed PDDs were compared to DDDs, and PDDs and DDDs were used to compare how closely the current prescribing practices matched with the recommendations from the literature on daily dosing of osteoporotic medications. Therefore, the PDD/DDD ratio was calculated based on standard dosing regimens. Given the use of fixed-dose therapies in osteoporosis management, values were expected to approximate 1.0.

### 2.4. Assessment of Medication Adherence

Medication adherence was assessed using the eight-item Morisky Medication Adherence Scale (MMAS-8), a validated self-reported tool for measuring patients’ medication-taking behavior. Use of the MMAS-8 in this study was authorized under an official license obtained from Morisky Medication Adherence Research, LLC, Las Vegas, NV, USA, prior to administration of the instrument (Certificate No. 1602-3937-6699-3794-8237). Each of the eight questions from this scale asks patients questions related to the manner in which the patients take their medication on a day-to-day basis; thus, total MMAS-8 scores can classify patients taking medication into three categories: Low, Medium, and High adherence. Medication adherence was assessed using the MMAS-8, with standard scoring thresholds applied: scores <6 were classified as low adherence, scores from 6 to <8 as medium adherence, and a score of 8 as high adherence. For analytical purposes, adherence was further dichotomized into adherent (score = 8) and non-adherent (score < 8) to facilitate regression analysis. The MMAS-8 was used as a self-reported measure of medication adherence based on versions available in the public domain. It was administered in a language understandable to the participants using a structured interview approach to ensure consistency in responses. In addition, patient knowledge and QoL were also assessed.

### 2.5. Assessment of Beliefs About Medicines

Patients’ beliefs regarding their osteoporosis medications were explored through the Beliefs about Medicines Questionnaire (BMQ), a validated instrument that assesses patients’ perceptions of medication necessity and concerns. This scale assesses how much obligation patients feel to continue taking medication in order to maintain quality of health, as well as concerns regarding the side effects and long-term effects of medication use. Based on the Necessity–Concerns framework, patients were categorized into four attitudinal groups as follows: Accepting (high necessity, low concerns), Skeptical (low necessity, high concerns), Indifferent (low necessity, low concerns), and Ambivalent (high necessity, high concerns). These categories were derived by comparing individual scores on the necessity and concern scales. BMQ scores were categorized based on the group mean to distinguish between relatively higher and lower belief levels within the study population, as there are no universally established cutoff values for these domains. This approach was used for comparative analysis within the sample.

### 2.6. Statistical Analysis

Data were analyzed using IBM SPSS Statistics version 29.0 (IBM Corp., Armonk, NY, USA) for descriptive statistics, between-group comparisons (Pearson χ^2^, Fisher’s exact test, Mann–Whitney U test), and standard logistic regression. Firth’s penalized maximum-likelihood logistic regression was performed in R version 4.4.1 (R Foundation for Statistical Computing, Vienna, Austria). A two-sided *p*-value < 0.05 was considered statistically significant. Univariate and multivariable logistic regression analyses were performed using Firth’s penalized maximum likelihood to identify factors associated with medication adherence. Firth’s method was chosen because three continuous predictors (BMQ-Specific Necessity, BMQ-Specific Concerns, and the Qualeffo-41 score) produced quasi-complete or complete separation against the dichotomised adherence outcome, under which standard maximum-likelihood estimates are not well defined. The multivariable Firth logistic regression model was constructed using an a priori, theory-driven approach. Candidate predictors, chosen on clinical grounds rather than by stepwise selection, were as follows: age (>60 vs. ≤60), sex, nationality, education, diabetes, hypertension, prior fracture, number of comorbidities (>2 vs. ≤2), number of concomitant medications (>2 vs. ≤2), BMQ-Specific Necessity (continuous), and BMQ-Specific Concerns (continuous). All candidates were entered simultaneously.

The Qualeffo-41 score, which showed complete separation, was excluded from the primary multivariable model. Multicollinearity among independent variables was quantified by variance inflation factors (VIF). Odds ratios (ORs) are reported with Wald 95% confidence intervals and two-sided *p*-values. Model discrimination was assessed by the area under the receiver-operating-characteristic curve (AUC, C-statistic), calibration by the Hosmer–Lemeshow goodness-of-fit test, and overall fit by Nagelkerke’s pseudo-R^2^. There were no missing data for the variables entered into the regression models.

### 2.7. Ethical Considerations

The study received ethical approvals from the Research and Ethics Committee of RAK College of Pharmacy (Approval No. RAKCOP/REC/2024-25/012) on 23 October 2024, the Research and Ethics Committee of RAK Medical and Health Sciences University (RAKMHSU-HEC-114-2024/25-PG-P) on 12 November 2024, and from the Ministry of Health and Prevention Research Ethics Committee—RAK Subcommittee (MOHAP/REC/2024/110-2024-PG-P) on 6 December 2024.

## 3. Results

### 3.1. Patients’ Characteristics

A total of 300 individuals suffering from osteoporotic disease were included in our study. The majority of patients were female, with a mean age of [70 y/o] (IQR: [63.0–76.0]). Most of the patients were older (≥50 y/o). The most prevalent co-morbidity amongst these patients were hypertension, and many of the patients had reported multiple co-morbidities. Many patients had a history of osteoporotic fractures, with vertebral fractures and non-vertebral fractures being the two most commonly reported types of fractures by these patients. Our study found generally diminished bone density, with a median T-score of −2.3 (IQR: −2.8 to +1.5), indicating a clinically significant burden of low bone mass at the time of the study. The reported T-score distribution was rechecked for accuracy. The observed range reflects variability in disease severity and treatment status, as some patients were included based on clinical diagnosis and may have demonstrated improved BMD values following therapy. Based on T-score classification, patients were divided into three clinically meaningful groups: osteopenia, osteoporosis, and severe osteoporosis. Many of the participants had sustained at least one fragility fracture prior to study entry, which reflects the real-world burden of the disease in this population. An overview of the demographic and clinical characteristics of the enrolled patients is provided Table 1.

**Figure 1:**
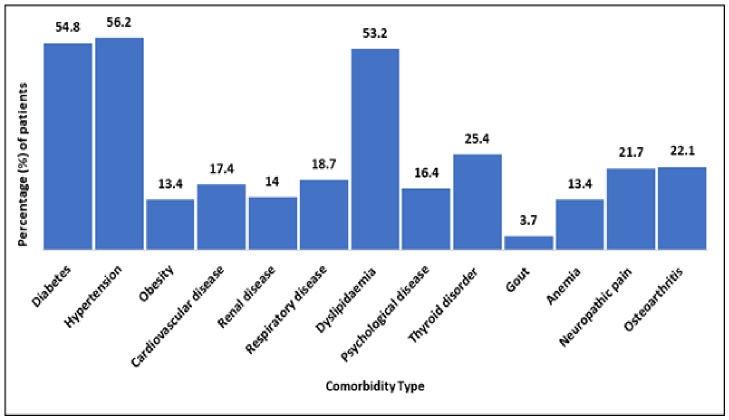
Types of Comorbidities.

Hypertension emerged as the most prevalent comorbidity among the study population, affecting 168 patients (56.2%), followed by diabetes mellitus in 137 patients (54.8%) and dyslipidemia in 159 patients (53.2%). The overall comorbidity profile of the study participants is illustrated in Figure 1.

### 3.2. Prescription Patterns

**Figure 2:**
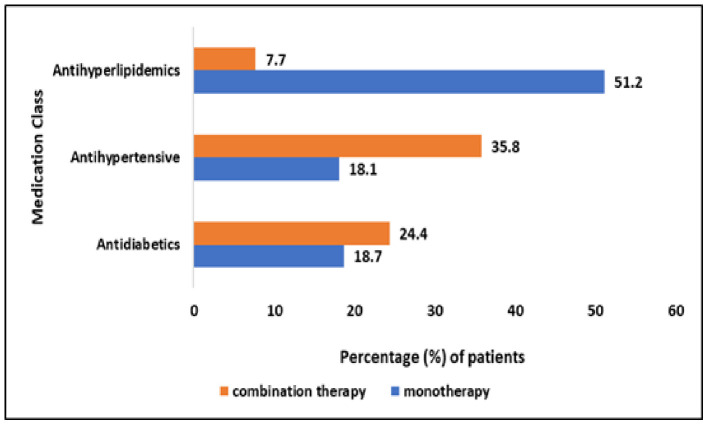
Concomitant Medications.

Anti-hyperlipidemic were the most commonly prescribed concomitant medications among the study population, with 153 patients (51.2%) receiving them as monotherapy and 23 patients (7.7%) as part of combination therapy. This was followed by antihypertensives, prescribed to 54 patients (18.1%) as monotherapy and 107 patients (35.8%) in combination regimens. The overall distribution of concomitant medications is illustrated in Figure 2.

The most prescribed medication was denosumab for the treatment of osteoporosis in our population (59% of participants) compared to three other drug types, romosozumab (31%), parathyroid hormone (4%), and bisphosphonates (2%). In our study, only a small minority (3%) of study participants did not receive any type of treatment for osteoporosis. The majority of participants received denosumab (*n* = 177; 59%) with a dose of (60 mg every 6 months; romosozumab (*n* = 92; 30.7%) with a dose of (105 to 210 mg monthly); teriparatide (*n* = 8; 4.7%) with a dose of 20 micrograms; and alendronate (*n* = 8; 2.7%) with a dose of either 10 mg or 70 mg weekly, respectively. Most frequently, participants received denosumab every 6 months and romosozumab once a month; participants who received teriparatide received it daily. For a complete overview of the monoclonal antibodies’ prescription patterns, their mechanisms and dosage are presented in Table 2.

The results of our PDD/DDD indicate that the drugs and dose prescribed for the treatment of osteoporosis at our study site were very close to the standard dosing and also to the WHO recommended doses. A slight difference was noted between the amount of drug that was actually prescribed to patients, and the amount need to be prescribed as per WHO reference. PDD was derived from the actual prescribed dose and administration frequency recorded in the medical charts and converted to an average daily dose before comparison with WHO-defined daily dose (DDD). Most medications demonstrated PDD/DDD ratios approximately equal to 1.0, indicating close alignment with standard dosing recommendations used in routine clinical practice; specifically: denosumab (DDD 0.33; PDD 0.33), romosozumab (DDD 7.0; PDD 7.0), teriparatide (DDD 20; PDD 20), and alendronic acid (DDD 10; PDD 10). Overall, our study shows a very high degree of agreement between the doses that were actually prescribed to patients, compared to WHO ATC/DDD reference doses; A summary of DDDs, PDDs, and PDD-to-DDD ratios is shown in Table 3.

### 3.3. Characteristics of Patients Stratified by Adherence

Based on MMAS-8 scores, patients were classified as non-adherent (*n* = 178; 59.3%) or adherent (*n* = 122; 40.7%) (Table 4). The median age was comparable between groups (70 years [IQR: 63–76] in the non-adherent group vs. 69 years [IQR: 63–76] in the adherent group; Mann–Whitney U = 10813; *p* = 0.951). No statistically significant differences were observed between adherent and non-adherent patients with respect to sex, nationality, prior fracture history, or the most commonly reported comorbid conditions (all *p* > 0.05). In contrast, adherence showed a clear relationship with medication beliefs. Adherent patients were predominantly observed in the high necessity/low concern group, indicating a strong association between patient beliefs and adherence compared with non-adherent patients (Table 4). Consistent with this pattern, an “Accepting” belief profile was the most common among adherent participants.

Comorbidity profiles were comparable between adherent and non-adherent patients. The prevalence of diabetes (42.7% vs. 50.8%; *p* = 0.166), hypertension (56.7% vs. 55.7%; *p* = 0.863), obesity (12.4% vs. 14.8%; *p* = 0.549), cardiovascular disease (19.7% vs. 13.9%; *p* = 0.198), chronic kidney disease (14.0% vs. 13.9%; *p* = 0.978), respiratory disease (18.5% vs. 18.9%; *p* = 0.945), dyslipidaemia (53.4% vs. 52.5%; *p* = 0.876), and psychological disorders (16.3% vs. 16.4%; *p* = 0.981) did not differ significantly between the two groups.

### 3.4. Factors Associated with Medication Adherence

In the univariate Firth penalized logistic regression (Table 5), none of the demographic or clinical variables showed a significant association with medication adherence. Adherence was not significantly related to age (OR 0.785; 95% CI 0.436–1.416; *p* = 0.421), gender (OR 1.049; 95% CI 0.501–2.196; *p* = 0.899), nationality (OR 0.987; 95% CI 0.574–1.696; *p* = 0.962), educational level (OR 1.075; 95% CI 0.676–1.707; *p* = 0.761), or marital status (OR 0.816; 95% CI 0.492–1.353; *p* = 0.430). In contrast, both belief domains were strongly associated with adherence when analysed by Firth penalized likelihood. Stronger necessity beliefs were significantly associated with greater adherence (OR = 220.58 per 1-unit increase; 95% CI: 14.02–3471.71; *p* < 0.001), while stronger concern beliefs were significantly associated with lower adherence (OR = 0.009 per 1-unit increase; 95% CI: 0.001–0.124; *p* < 0.001). The large magnitudes reflect the near-perfect separation observed between adherent and non-adherent groups on these continuous scales; Firth penalization provides finite, interpretable estimates under these conditions.

In the multivariable Firth logistic regression (Table 6), stronger necessity beliefs remained positively associated with adherence, although the effect did not reach statistical significance after adjustment (OR = 23.07 per 1-unit increase; 95% CI: 0.67–792.71; *p* = 0.082), while stronger concern beliefs were independently and significantly associated with reduced adherence (OR = 0.033 per 1-unit increase; 95% CI: 0.003–0.355; *p* = 0.005).

A higher comorbidity burden remained significantly associated with lower adherence. Patients with more than two comorbidities had substantially reduced odds of adherence compared with those with two or fewer comorbidities (OR = 0.076; 95% CI: 0.008–0.688; *p* = 0.022). After adjustment for belief domains and the comorbidity burden, the association between the number of concomitant medications and adherence was no longer statistically significant (OR = 2.379; 95% CI: 0.327–17.32; *p* = 0.392), suggesting that once disease burden and medication beliefs are accounted for, the independent contribution of medication count is small and imprecisely estimated in this sample.

The Qualeffo-41 quality-of-life score was not included in the primary multivariable model because it exhibited complete separation against the adherence outcome, which destabilizes estimation even under Firth penalization when other near-separating predictors are present. A sensitivity analysis retaining Qualeffo-41 is provided in Supplementary Table S1.

## 4. Discussion

Two persistent real-world problems are highlighted by osteoporosis research, namely the following: patients do not typically take their medicines properly, and many face some practical barriers/challenges to get or continue their treatment. Despite the wide availability of effective strategies and standard treatment guidelines for the management of osteoporosis, a major gap between adherence and continuity of patient care still persists. Since osteoporosis leads to the majority of fragile fractures, the risk is especially alarming in older adults with chronic conditions and taking complex medications regimens that require close monitoring. In this setting, the risk that patients will miss doses, stop treatment early, or develop treatment fatigue is high [8]. In this context, our study explored the utilization patterns of drugs, adherence to prescribed medications, and well-being of patients with osteoporosis who were being treated at a secondary care facility in the UAE. Several findings were consistent with published epidemiology data. First, the patients studied were mostly women, which supports the most well-known higher rate of osteoporosis in women after menopause [9]. This finding is consistent with the evidence that there is a lack of estrogen which causes rapid bone loss after menopause and thereby increases the risk of fractures and need for long-term preventative therapy [10].

Secondly, the large sample in our study consisted primarily of older adults, which is consistent with the findings in the literature of population-based studies showing increased risk of bone mineral density loss with increasing age and, consequently, the associated increased risk of fracture. These findings validate that osteoporosis care in secondary care settings is usually directed to older women who may benefit the most from specialized education, compliance assistance, and optimization of their therapy when they are experiencing multiple diseases at the same time and/or are prescribed with multiple medications [11].

Our study participants had a larger percentage of Emirati nationals than others, which is likely a reflection of the study site’s catchment population. This study also provided an opportunity to more closely examine the characteristics of osteoporosis in Emirati patients than has been possible with other nationality groups. Participants in this study reported a family history of osteoporosis in only 0.7% of cases. This suggests an overall lack of awareness about the potential for family members to be at risk for developing osteoporosis, a lack of recognition among family members of being diagnosed with osteoporosis, or incomplete or uncertain reporting by study participants [12]. Underreporting of a family history of osteoporosis may lead to misclassification of patients and may weaken any correlation between a positive family history of osteoporosis and osteoporosis-related outcomes in this study population. Approximately 50% of study participants had received some form of education, which generally is positively correlated with greater health literacy, including better adherence to prescribed medications according to the literature [13]. In this study, no statistically significant association was observed between education level and medication adherence. This finding suggests that knowledge alone may not be sufficient to improve adherence, and that psychosocial factors such as medication beliefs may play a more important role in shaping adherence behavior. However, this finding should be interpreted with caution, as it may be influenced by sample characteristics and the use of self-reported adherence measures. Therefore, factors other than formal education may relate to adherence behavior in this population. Some examples of factors that may affect adherence include culturally-based beliefs regarding osteoporosis and its treatments; the quality of communication between patients and healthcare providers; differences in access to, and ability to navigate, the healthcare system; and logistical barriers to obtaining or continuing medications.

The results of this study indicate that both the frequently mentioned factors (e.g., poor health literacy and lack of understanding due to education) are influencing people not to take their medications. Other factors are specifically related to the circumstances/attributes in which someone’s ongoing use of medication occurs (e.g., their respective local cultures, the way their health care system works, patient provider interactions, etc). These must be considered when developing strategies aimed at improving medication adherence within the Gulf Region. To this end, conducting additional quantitative and qualitative studies will help researchers more fully identify all of the driving forces behind a given patient’s non-compliance while simultaneously providing insights towards designing potentially suitable culturally relevant interventions to increase adherence in GULF populations.

The majority of participants in this study were unemployed. This could be explained by older age characteristics of the group and the functional burden of osteoporosis and other health related conditions. Multimorbidity was frequently observed, and a substantial portion of patients reported diabetes, dyslipidemia, and hypertension, which made their overall management more challenging [14]. Care for osteoporosis in older adults can be challenging; multimorbidity and polypharmacy are repeatedly identified as key factors to this complexity. When patients have several comorbid conditions, they often require multiple concurrent medicines, which increases the risk of drug–drug interaction and significantly increases the total treatment burden [7]. Although a descriptive trend suggested relatively better adherence among younger patients, this association was not statistically significant in our analysis and should be interpreted with caution. Our findings support the previous evidence, that multimorbidity and polypharmacy may interfere with osteoporosis care by making medication therapy more complex. Therefore, patients may be more likely to discontinue therapy, experiencing negative outcomes, potentially reducing overall health outcomes.

In the present study, denosumab (a RANKL inhibitor) was the most frequently prescribed osteoporosis medication [15]. Denosumab is administered as a subcutaneous injection every six months, a schedule that may be convenient for patients and easier to implement in clinical follow-up. Its established effectiveness in reducing fracture risk may also contribute to clinicians’ preference for this therapy in routine practice [16]. The six-monthly subcutaneous dosing schedule may be more practical for patients and simplify follow-up in routine care. Its well-documented meaningful fracture prevention may also contribute to clinicians’ preference to use denosumab in everyday clinical settings [17]. The second most frequently ranked drug was romosozumab. This sclerostin inhibitor combines both anabolic and antiresorptive actions. The substantial prescribing of romosozumab may reflect its growing role in therapy for individuals at elevated fracture risk and the gradual adoption of newer agents among clinicians in routine clinical practice [18]. Teriparatide and alendronate were used less often, possibly due to differences in treatment preference (injectable versus oral therapy), cost, access, and individual clinical characteristics that may influence prescribing decisions. Nearly all the study participants in our study received at least one osteoporosis medication, which reflects its widespread compliance with the standard recommended guidelines [19].

In general, the analysis of drug use indicated a high degree of agreement between WHO reference dosing and actual prescribing in the clinical practice. Most of the drugs showed PDD/DDD ratios of 1.0, demonstrating that the daily doses prescribed were in good agreement with the WHO ATC/DDD benchmarks [20]. The result of our study indicates that the prescription practice at the study location was largely rational and in agreement with established protocols, signifying the implementation of osteoporosis management. The close agreement between the prescribed drug dose and the reference standard indicates that the care in our setting was delivered in accordance with established guidelines [21].

Supportive management was also commonly used in the management of osteoporosis. The guidelines recommend the use of calcium carbonate, alfacalcidol, and vitamin D, as they are very beneficial to bone health. Therefore, it can be used as a secondary therapy for the management of osteoporosis. The care plan also includes the management of pain. Celecoxib and meloxicam, which are non-steroidal anti-inflammatory drugs (NSAIDs), were recommended to a large number of patients. This could be a suggestion that the participants had a high prevalence of chronic pain, which could be due to the consequence of old fractures and degenerative joint disease, as seen in osteoporosis patients [22]. Diclofenac gel and ketoprofen gel (topical painkillers), were also commonly recommended. This could be, again, an indication that the prescriber had a bias towards topical pain management, possibly to avoid the side effects associated with NSAIDs [23].

In addition to the prevention of fracture, topical treatments like glucosamine and Reparil gel were also used. This might suggest a more general supportive approach that aimed to reduce the symptoms while minimizing the pain and improving the mobility and quality of the patient’s life [24].

Adherence was measured using the MMAS-8 tool. Our study results showed that adherence to the treatment regimen applied to less than half of the participants. This clearly shows that adhering to the treatment regimen over a long period is still challenging. Our study results showed that younger patients with sufficient vitamin D levels strongly adhered to osteoporosis treatment. This is because younger patients may face fewer challenges in their day-to-day activities. Furthermore, they may be more motivated to adhere to the treatment regimen over a long period. Adequate vitamin D levels might also clearly suggest that the patient is more health-conscious. Adherence to osteoporosis treatment is also associated with QoL across various domains. Our research closely agrees with the findings of Ahmed et al. (2023), that lower fracture risk is strongly associated with improved adherence [25]. The Beliefs about Medicines Questionnaire (BMQ) was used to examine the perceptions of the patients specifically in terms of necessity and concerns about the treatment of osteoporosis. The belief patterns were found to be highly related to medication adherence and patient outcome. The patients were grouped according to their beliefs, and the “Accepting” group, which is defined by the combination of high necessity/low concerns, had the best medication adherence and health outcomes.

Our findings are in accordance with the results drawn by Shahin et al. (2019), who proposed that patients’ adherence to medication in long-term diseases depends solely on patients’ perceived need for medication and fear of possible adverse events [26]. Contrary to our findings, Rasool et al. (2023) did not find any noteworthy link between disease perceptions and medication compliance in their study population [27]. The differences in findings might be credited to the differences in methodologies and different populations groups. These conclusions highlight the necessity for additional investigation in this field, especially in the Gulf region. The concerns of the patients about their health may be influenced by their understanding of osteoporosis and the treatment possibilities available. Overall, high level of understanding might enhance compliance by enabling patients to know the advantages of drug treatment and reduce concerns about their potential risks. Our findings suggested that patients’ compliance with medication was generally higher if patients’ need for osteoporosis medication was higher than their concerns about it. On the other hand, patients’ compliance with medication was lower if patients’ concerns outweighed their needs. Our analysis revealed that, although a small number of patients had a high level of knowledge about osteoporosis and treatment options, a large number had a poor level of knowledge about disease and treatment options. Sociodemographic and psychosocial factors such as employment status, educational level attained, and beliefs regarding medications (accepting and non-accepting attitudes) appeared to play a major role in these trends. In summary, our results support the findings of the prior study conducted by Thakur et al., which reported that there are many patients who lack sufficient knowledge regarding osteoporosis and that pharmacist-led educational and training programs should be implemented to improve long-term management and increase patient compliance [28].

The correlation analysis in this study showed that adherence had a positive correlation with necessity beliefs, whereas concern beliefs were negatively correlated with adherence. These results emphasize the complex interplay among patient convictions and compliance. Our findings reinforce the importance of the biopsychosocial model in the management of patients with osteoporosis, underscoring the need for a comprehensive approach that considers biological aspects alongside psychological and social factors. A clear connection was found between medication adherence and patients’ perceptions. Patients who adhered to their medications held stronger beliefs about its necessity, highlighting that valuing osteoporosis medication is crucial for maintaining compliance. In contrast, concerns about potential side effects were associated with non-adherence, highlighting how medication-related concerns affect illness management. Collectively, these outcomes support the notion that a biopsychosocial approach should be implemented in the management of osteoporosis. The best outcomes cannot be achieved with pharmacotherapy alone but also require ongoing support, taking into consideration the psychological and social factors that influence patient behavior. Such an approach may improve patient compliance and raise the overall standard of osteoporosis care.

Our results indicated a clear link between patients’ perceptions of their osteoporosis therapy and their adherence to medication. Every patient who followed the treatment had high necessity and low concern beliefs, whereas most non-adherent patients were categorized as “Skeptical” or “Indifferent.” This pattern aligns with the necessity–concerns framework proposed by Horne et al. (2020), which suggests that the balance between perceived treatment necessity and fear of side effects is a stable predictor of adherence behavior [29].

Our findings are consistent with those of Yeam et al. (2018), who suggested that patients who believe strongly in the importance of osteoporosis medications are more likely to adhere to them [30]. Collectively, these findings highlight that in addition to clinical factors, patients’ perceptions and attitudes towards treatment play an important role in adherence. Emphasizing both the cognitive factor (reinforcing perceived necessity) and the emotional factor (addressing worries and fears of adverse effects) through personalized counselling may play an important role in patient adherence.

Although higher knowledge scores appeared to show a trend towards better adherence, this association was not statistically significant and should be interpreted with caution. Consistent with our results, Idrissi Zaki et al. (2022) noted that a poor grasp of osteoporosis risks and outcomes was associated with higher rates of non-compliance [31].

In the present study, patients usually reported lower necessity beliefs and heightened concerns about their osteoporosis treatments, which were assessed using BMQ scale. These beliefs were strongly associated with medication adherence. Participants identified as “Accepting” (high necessity and low concern) demonstrated notably better adherence than those in the other belief categories. A significant positive relationship was found between osteoporosis knowledge and BMQ-Necessity, indicating that patients with a better understanding of osteoporosis were more likely to view their medications as essential. In contrast, osteoporosis awareness showed no notable relationship with BMQ-Concerns [32]. This suggests that improving comprehension may strengthen perceived necessity but alone may be insufficient to address concerns about adverse effects or prolonged risks. This viewpoint aligns with the necessity–concerns framework described by Unni et al. (2022), emphasizing perceived medication necessity as an essential element influencing adherence behavior [33].

## 5. Limitations

This single-centered study took place at a secondary care hospital in the UAE. Data collection mostly depended on structured questionnaire, patient case records, and laboratory reports. This might have contained missing or incomplete information. As this was a single-center study conducted in a secondary care hospital, the study population may not fully represent the broader UAE population, and there is a potential for selection bias. Because of the cross-sectional design, this study can identify associations between knowledge, beliefs, and medication adherence, but it cannot establish causal or temporal relationships among these variables. These findings should be interpreted as associative rather than causal, given the cross-sectional nature of the study. Furthermore, the study did not consider possible differences in osteoporosis treatment approached between private and public sectors, or among physicians with varying specialties. BMQ and MMAS-8 are validated measure, yet they rely on self-reported tools. Therefore, when evaluating adherence and patient beliefs it can be influenced by recall and social desirability bias. While the PDD/DDD ratio provides insight into prescribing patterns at the population level, it may not fully reflect individualized clinical appropriateness, as patient-specific factors were not incorporated into this measure. Unfortunately, to gain a better understanding and delve deeper into the economic consequences of prescribing Denosumab, Romosozumab, or other advanced osteoporosis treatments, this study did not focus on cost-effective analysis. Despite the presence of comorbidities and polypharmacy, the study did not thoroughly examine the risks of drug–drug interactions. This may be important for older adults taking multiple medications and warrants further investigations.

The findings of our study may primarily reflect prescribing patterns and patient behaviors within similar healthcare settings. Differences in healthcare delivery models, patient populations, and access to specialized services in primary care, private, or tertiary care settings may influence medication prescribing, adherence, and patient perceptions. Overall, the use of standardized and validated instruments and a structured data collection approach may help mitigate some of these limitations. Thus, the generalizability of these results to other healthcare settings within the UAE or across Gulf Cooperation Council (GCC) countries should be interpreted with caution. Future multicenter studies across diverse healthcare settings are warranted to validate and extend these findings.

## 6. Conclusions

This study provides real-world evidence on prescribing patterns, medication adherence, and quality-of-life (QoL) outcomes in patients receiving anti-osteoporotic medications, including monoclonal antibodies (mAbs), and is among the first from the United Arab Emirates to report these outcomes in this setting. Denosumab and romosozumab accounted for 89.7% of prescribed mAb therapies, reflecting alignment with current clinical practice patterns. Prescribing appeared appropriate in terms of dosage, as shown by PDD/DDD ratios close to 1.0, indicating alignment between prescribed doses and WHO ATC/DDD standards.

Despite continuing treatment, patients reported moderate decrements in QoL across all Qualeffo-41 domains, suggesting that the burden of osteoporosis remains substantial even among those receiving treatment. Adherence rates were not optimal, as follows: 59.3% of participants were classified as low adherers, whereas only 11.3% exhibited high adherence. These findings have implications for healthcare professionals, including pharmacists, in supporting medication adherence. Demographic factors such as age, sex, nationality, and education did not demonstrate significant independent predictive value for adherence.

Among clinical factors, a higher comorbidity burden was independently associated with lower adherence, whereas the number of concomitant medications was not an independent predictor after adjustment for medication beliefs and comorbidity burden. Patient-centered factors such as beliefs about medication, perceived necessity, and concerns had a greater impact on adherence behavior than routine demographic or clinical characteristics. Psychosocial factors also had a greater impact on adherence than osteoporosis knowledge alone, as follows: stronger necessity beliefs were associated with better adherence, while greater concerns were tied to reduced adherence, highlighting the important role of patient perceptions in maintaining long-term therapy. These findings should be interpreted cautiously given the study’s cross-sectional nature; observed associations do not imply causation. Nevertheless, they suggest that multimorbidity, together with patients’ beliefs about their medications, poses meaningful barriers to maintaining long-term osteoporosis treatment regimens.

## 7. Recommendations

Despite that many participants confirmed an adequate understanding of osteoporosis, this awareness by itself did not lead to notably improved adherence. This shows that although education is important, tackling patients’ belief systems and emotional reactions to treatment might have greater impact on adherence than providing information alone. Thus, managing osteoporosis, especially in patients undergoing anti-osteoporotic therapy, must incorporate behavioral strategies and belief-based approaches along with pharmacological optimization and standard patient education. Additional multicenter and longitudinal studies are required to validate these results, elucidate causal mechanisms, and assess the cost-effectiveness of interventions aimed at improving adherence. Future efforts should focus on developing targeted strategies to improve adherence, tackle medication-related beliefs, and reduce difficulties associated with polypharmacy. Together, these actions are critical for enhancing patient-centered care and advancing outcomes for osteoporosis patients receiving anti-osteoporotic medications, including monoclonal antibodies.

## Figures and Tables

**Table 1:** Socio-demographic and clinical characteristics of the study population (*n* = 300).

Variable	N (%)/Median	95% Confidence Interval/IQR
Age, year (median/IQR)	70	63–76
Age group, years		
≤60	55 (18.3)	14.4–23.1
>60	245 (81.7)	76.9–85.6
Gender		
Female	266 (89)	84.6–91.8
Male	34 (11)	7.9–15.0
Nationality		
Emirati	229 (76.3)	71.2–80.8
Non-Emirati	71 (23.7)	19.2–28.8
Education status		
Educated	163 (54.4)	48.7–59.9
Not educated	137 (45.6)	39.8–51.0
Fracture history	96 (32.0)	27.0–37.5
Comorbidities burden, median (IQR)	1.0	0.0–3.0
One to two	71 (23.7)	19.2–28.8
More than two	229 (76.3)	71.2–80.8
Concomitant medications (count)		
One to two concomitant Meds	64 (21.3)	17.1–26.3
More than two concomitant Meds	236 (78.8)	73.7–82.9
Polypharmacy		
Less than 5 medication	162 (54.0)	48.3–59.6
5 or more medication	138 (46.0)	40.4–51.7
Osteoporosis pharmacotherapy class		
RANKL Inhibitor	177 (59.0)	53.4–64.4
Sclerostin Inhibitors	92 (30.7)	25.7–36.1
bisphosphonate	8 (2.7)	1.4–5.2
PTH analogues	8 (4.7)	2.8–7.7
No medication	9 (3.0)	1.6–5.6
Key Labs, median (IQR)		
T-score	−2.3	−2.8–1.5
Serum calcium	2.3	2.2–2.4
Serum vitamin D	71.5	55.0–92.5

**Table 2:** Monoclonal antibodies, their mechanisms and dosage regimen.

Osteoporosis Medication	Mechanism of Action	Dose	Frequency	N (%) (*n* = 300)
Denosumab	RANKL Inhibitor	60 mg	Q6M	177 (59.0)
Romosozumab	Sclerostin Inhibitors	105 mg 210 mg	Q1M	92 (30.7)
Teriparatide	PTH analogues	20 mcg	OD	8 (4.7)
Alendronate	Bisphosphonate	10 mg 70 mg	Q 7D	8 (2.7)

**Table 3:** Defined daily doses and prescribed daily doses of osteoporosis medication for the study population.

Osteoporosis Medications	Dosing Regimen Prescribed	WHO DDD	Calculated Mean PDD	PDD/DDD
Denosumab	60 mg SC every 6 months	0.33 mg/day	0.33 mg/day	1.00
Romosozumab	210 mg SC monthly	7 mg/day	7.00 mg/day	1.00
Teriparatide	20 mcg SC daily	20 mcg/day	20 mcg/day	1.00
Alendronic acid	70 mg orally weekly	10 mg/day	10.0 mg/day	1.00

**Table 4:** Demographic and clinical characteristics stratified by adherence (MMAS-8).

Variable	Adherence Status		*p*-Value *
Variable	Non-Adherent (*n* = 178)	Adherent (*n* = 122)	
Age, year [median/IQR]	70 (63–76)	69 (63–76)	U = 10,813; *p* = 0.951
≤60	30 (16.9%)	25 (20.5%)	0.424
>60	148 (83.1%)	97 (79.5%)	
Gender			0.875
Male	20 (11.2%)	13 (10.7%)	
Female	158 (88.8%)	109 (89.3%)	
Nationality			0.972
Emirati	136 (76.4%)	93 (76.2%)	
Non-Emirati	42 (23.6%)	29 (23.8%)	
History of fracture			0.992
Yes	57 (32.0%)	39 (32.0%)	
No	121 (68.0%)	83 (68.0%)	
Marital status			0.431
Married	130 (73.0%)	84 (68.9%)	
Not-married	48 (27.0%)	38 (31.1%)	
Education			0.942
Yes	97 (54.5)	67 (54.9)	
No	81 (45.5)	55 (45.1)	
Employment			0.394
Yes	35 (19.7%)	29 (23.8%)	
No	143 (80.3%)	93 (76.2%)	
No. of comorbidities, median [IQR]	4 (3–5)	4 (3–5)	U = 10,059.5; *p* = 0.279
One to two	40 (22.5)	31 (25.4)	0.556
More than two	138 (77.5)	91 (74.6)	
Concomitant medications, median [IQR]	4 (3.0–6.0)	4 (3.0–6.0)	U = 10,643; *p* = 0.771
One to two concomitants	39 (21.9)	25 (20.5)	0.768
More than two concomitants	139 (78.1)	97 (79.5)	
Polypharmacy			0.977
Less than 5 medication	96 (53.9)	66 (54.1)	
5 or more medication	82 (46.1)	56 (45.9)	
OKAT (knowledge), median [IQR]	9 (8–10)	8 (7–10)	U = 9776; *p* = 0.143
Poor knowledge	91 (51.1)	70 (57.4)	
Good knowledge	87 (48.9)	52 (42.6)	
BMQ Necessity			<0.001
Low necessity	158 (88.8)	0 (0.0)	
High necessity	20 (11.2)	122 (100.0)	
BMQ Concern			<0.001
Low concern	64 (36.0)	122 (100.0)	
High concern	114 (64.0)	0 (0.00)	
BMQ Attitudinal			
Accepting	20 (11.2)	122 (100.0)	
Skeptical	114 (64.0)	0 (0.0)	
Indifferent	44 (24.7)	0 (0.0)	

* Pearson Chi-square test, Fisher’s exact test, Monte Carlo test, Mann–Whitney U test as applicable. IQR: Interquartile range, BMQ: Beliefs about Medicines Questionnaire; OKAT: Osteoporosis Knowledge Assessment Tool.

**Table 5:** Univariate logistic regression analysis of factors associated with medication adherence.

Variable	Category/Contrast	OR *	95% CI	*p*-Value
Age, years	>60 vs. ≤60	0.785	0.436–1.416	0.421
Gender	Female vs. Male	1.049	0.501–2.196	0.899
Nationality	Emirati vs. Non-Emirati	0.987	0.574–1.696	0.962
Education	Educated vs. Non-educated	1.075	0.676–1.707	0.761
Marital status	Married vs. Not-married	0.816	0.492–1.353	0.430
Diabetes	Present vs. Absent	1.384	0.871–2.199	0.168
Hypertension	Present vs. Absent	0.960	0.603–1.527	0.862
History of fracture	Present vs. Absent	1.000	0.610–1.638	0.999
No. of comorbidities	>2 vs. ≤2	0.849	0.496–1.455	0.552
No. of concomitant meds.	>2 vs. ≤2	1.083	0.615–1.904	0.783
OKAT knowledge	Good vs. Poor	1.284	0.808–2.042	0.290
Polypharmacy	≥5 vs. <5 meds	0.994	0.626–1.578	0.979
BMQ-Specific Necessity	per 1-unit increase	220.6	14.01–3471.7	<0.001
BMQ-Specific Concerns	per 1-unit increase	0.009	0.001–0.124	<0.001

* Firth logistic regression, Qualeffo-41 score is not reported in the univariate regression owing to complete separation against the adherence outcome. OKAT: Osteoporosis Knowledge Assessment Tool.

**Table 6:** Multivariate logistic regression model for factors associated with medication adherence.

Variable	Category/Contrast	aOR ^#^	95% CI	*p*-Value
Gender	Female vs. Male	0.375	0.041–3.415	0.384
Education	Educated vs. Non-educated	2.707	0.594–12.34	0.198
Age, years	>60 vs. ≤60	1.390	0.205–9.425	0.736
Nationality	Emirati vs. Non-Emirati	0.716	0.143–3.594	0.685
Diabetes	Present vs. Absent	0.493	0.091–2.671	0.412
Hypertension	Present vs. Absent	4.575	0.711–29.43	0.109
History of fracture	Present vs. Absent	0.876	0.173–4.446	0.873
No. of comorbidities	>2 vs. ≤2	0.076	0.008–0.688	0.022
No. of concomitant meds.	>2 vs. ≤2	2.379	0.327–17.32	0.392
BMQ-Specific Necessity	per 1-unit increase	23.07	0.671–792.7	0.082
BMQ-Specific Concerns	per 1-unit increase	0.033	0.003–0.355	0.0049

Firth logistic regression, ^#^ *n* = 300 (events = 122); Events Per Variable (EPV) = 11.09; AUC = 0.997; Nagelkerke R^2^ = 0.944; Hosmer–Lemeshow χ^2^ = 3.134, *p* = 0.926; max VIF = 5.80 (BMQ-Specific Necessity).

## Data Availability

The data presented in this study are available on request from the corresponding author. The data are not publicly available due to privacy and confidentiality concerns associated with patient data.

## Supplementary Materials

The following supporting information can be downloaded at: https://www.mdpi.com/article/10.3390/healthcare14091201/s1, Table S1: Sensitivity analysis: multivariable Firth model including Qualeffo-41.
