# Supplementary material for: Real-World Osteoporosis Pharmacotherapy in the UAE: Prescribing Trends, Adherence, and Patient Beliefs
Source: Healthcare (Basel). 2026 Apr 29;14(9):1201. doi: 10.3390/healthcare14091201 (PMC13163328; doi:10.3390/healthcare14091201)
Supplement: Supplementary file 1 [file healthcare-14-01201-s001.zip › healthcare-4189923-supplementary.pdf]

Supplementary Table:

Supplementary Table S1 — Sensitivity analysis: multivariable Firth model including Qualeffo-41

Variable	Contrast	aOR (Firth)	95% CI	p
Gender	Female vs Male	0.501	0.037 – 6.683	0.601
Education	Educated vs Non-educated	4.040	0.360 – 45.31	0.258
Age, years	>60 vs ≤60	2.389	0.188 – 30.39	0.502
Nationality	Emirati vs Non-Emirati	0.515	0.057 – 4.663	0.555
Diabetes	Present vs Absent	0.139	0.011 – 1.825	0.133
Hypertension	Present vs Absent	3.408	0.298 – 38.94	0.324
History of fracture	Present vs Absent	0.586	0.052 – 6.603	0.665
No. of comorbidities	>2 vs ≤2	0.298	0.024 – 3.755	0.349
No. of concomitant medications	>2 vs ≤2	4.022	0.335 – 48.34	0.273
BMQ-Specific Necessity	per 1-unit increase	0.942	0.022 – 40.57	0.975
BMQ-Specific Concerns	per 1-unit increase	21.92	0.276 – 1740.54	0.167
**Qualeffo-41 score**	per 1-unit increase	**0.014**	**0.001 – 0.174**	**<0.001**

n = 300. Events-per-variable = 10.2. AUC = 1.000. Maximum VIF = 34.20 (Qualeffo-41). Qualeffo-41 exhibits complete separation against the adherence outcome (non-adherent range 39.8–73.2; adherent range 14.1–39.7, no overlap). When retained in the model, it dominates the fit and destabilizes the other coefficient estimates, including the two BMQ domains whose directions invert relative to the primary model; this model is presented only for transparency and should not be interpreted as the primary result.
